# Development and validation of a self-administered questionnaire measuring essential knowledge in patients with rheumatoid arthritis

**DOI:** 10.1007/s00296-022-05090-8

**Published:** 2022-04-07

**Authors:** Malory Rodère, Bruno Pereira, Martin Soubrier, Françoise Fayet, Muriel Piperno, Béatrice Pallot-Prades, Sophie Pouplin, Guy Baudens, Jean-David Cohen, Pascal Coquerelle, Laurent Grange, Christelle Sordet, Sonia Tropé, Laure Gossec, Catherine Beauvais

**Affiliations:** 1 Rheumatology Department, Centre Hospitalier Universitaire Gabriel Montpied, Clermont-Ferrand, France; 2 Biostatistics Unit, Centre Hospitalier Universitaire Gabriel Montpied, Clermont-Ferrand, France; 3 Rheumatology Department, Centre Hospitalier Universitaire Lyon-Sud, Lyon, France; 4 Rheumatology Department, Centre Hospitalier Universitaire de Saint Etienne, Fondation partage et vie, Centre médical de l’Argentière, Hôpital Bellevue, Saint Etienne, France; 5 Rheumatology Department, Hôpital Universitaire de Rouen, Rouen, France; 6 Cabinet médical, Valenciennes, France; 7 Rheumatology Department, Centre Hospitalier Universitaire de Montpellier, Montpellier, France; 8 Rheumatology Department, Centre Hospitalier, Béthune, France; 9 Rheumatology Department, Centre Hospitalier Universitaire Grenoble Alpes, Echirolles, France; 10 Rheumatology Department, Hôpitaux Universitaires de Strasbourg, Strasbourg, France; 11 Association Nationale de Défense contre l’Arthrite Rhumatoïde, Paris, France; 12 Sorbonne Université, INSERM, Institut Pierre Louis d’Epidémiologie et de Santé Publique, Paris, France; 13 Rheumatology, Pitié Salpêtrière Hospital, APHP, Sorbonne Université, Paris, France; 14 Rheumatology Department, Sorbonne Université, Centre Hospitalier Universitaire Saint Antoine, Assistance Publique Hôpitaux de Paris, APHP, Paris, France

**Keywords:** Rheumatoid arthritis, Patient education, Patient therapeutic education, Knowledge questionnaire, Educational needs

## Abstract

To develop and validate a questionnaire assessing patient knowledge in rheumatoid arthritis (RA). Knowledge considered essential for patients with RA was identified through a series of Delphi rounds among rheumatologists, health professionals (HPs), patients, and then reformulated to construct the knowledge questionnaire. Cross-sectional multicenter validation was performed in 12 rheumatology departments to assess internal validity (Kuder–Richardson coefficient), external validity, acceptability, reproducibility (Lin’s concordance correlation coefficient) and sensitivity to change (difference in total score before and after patient education sessions). Associations between patient variables and knowledge levels were evaluated. RAKE (RA Knowledge questionnairE) is a self-administered 45-item questionnaire scored 0–100, with a 32-item short-form survey assessing knowledge of disease, comorbidity, pharmacological treatments, non-pharmacological treatments, self-care and adaptative skills. Of 130 patients included in the validation study, 108 were women. Acceptability was good with < 5% missing data. Internal validity coefficient was 0.90. Mean (standard deviation) long-form score was 72.8 ± 17.8, with lower scores in comorbidity and self-care and higher scores in adaptive skills. Reproducibility was good (0.86 [0.80; 0.92]). RAKE score was positively correlated with the patients’ level of education and the HPs’ opinion on the patients’ knowledge. RAKE score showed good sensitivity to change: 66.8 ± 16.4 then 83.8 ± 12.7, representing a hedges effect size of 1.14 [95% CI 0.73; 1.55]. RAKE is an updated questionnaire assessing essential knowledge for patients with RA to enhance self-management according to current guidelines and the patients’ perspective. RAKE can usefully inform patient education interventions, routine care and research.

**Supplementary Information:**

The online version contains supplementary material available at 10.1007/s00296-022-05090-8.

## Introduction

According to international guidelines [1–3], treatment decisions in rheumatoid arthritis (RA) should be made by physicians and patients through a shared decision-making process taking into account the patient’s values, preferences, and comorbidities. Patient education aims to enable patients and their family members to acquire the skills they need to manage life with their condition [4]. The European Alliance of Associations for Rheumatology (EULAR) advocates patient education as an integral part of standard care for people with inflammatory arthritis [5], to allow them to develop self-care and coping skills [5–7]. Patient education includes a wide range of activities based on a planned interactive process through face-to-face or group sessions or online offerings [5] that accommodate patient’s needs and values [8, 9].

Although patient education is not limited to knowledge, assessing patient knowledge is part of the educational process and may be carried out by means of questionnaires.

Several knowledge questionnaires (KQs) are available in the literature. The Patient Knowledge Questionnaire, which was developed in 1991 and validated again in 2004 [10, 11] and the Knowledge Questionnaire, which dates from 1997 [12], have both been used in several studies [14–17]. However, they were constructed before the era of targeted disease-modifying anti-rheumatic drugs (DMARDs). Their content, mostly focused on knowledge of disease, is not (at this time) in keeping with recent recommendations for RA management and patient needs [1–3, 13], particularly in terms of pharmacological treatment and strategy to remission, pain management and coping skills [13], comorbidities [18] and DMARD safety [19]. More recently, a 13-item questionnaire called, the rheumatoid arthritis knowledge assessment scale (RAKAS) was developed in Pakistan, but it has not been widely assessed or validated [20]. Other questionnaires specifically consider knowledge of pharmaceutical treatments such as methotrexate [21, 22] and biological DMARDs (bDMARDs) [23] but do not allow an overall assessment of patient knowledge.

To address this gap, the aim of this work was to construct and validate a generic KQ for RA patients for use in routine care and research.

## Methods

Methodological guidelines for the development of questionnaires were applied [24, 25].

### Construction of the questionnaire

The questionnaire was developed in three steps.

First, 90 knowledge items were extracted from published knowledge questionnaires [10, 12] or unpublished questionnaires commonly used in France. The items were used in a Delphi process including rheumatologists, health professionals (HPs) and patients with the objective to identify knowledge considered relevant for RA patients. The Delphi rounds involved 107 participants from 13 multidisciplinary teams across France. The first Delphi round enlarged the list to 322 items. To ensure a reasonable completion time, the second round was performed in two parts because of the large number of items to be selected. Among the 69 key knowledge items selected, 36 (52%) were not present in the existing published KQs or were modified, with fewer items for knowledge of disease and more items for treatment strategy and DMARDs. [13].

In a second step, a final Delphi round selected a list of 45 items for this study: 32 items considered essential were selected by more than 66% and 13 items considered useful were selected by more than 50% of participants. Two rheumatologists and a rheumatology nurse constructed the first version of the KQ, with each question referring to a selected item in the list. Response options for each question were True, False, and I do not know.

The questionnaire was reformulated during a face-to-face consensus-finding meeting between three rheumatologists, a rheumatology nurse and a patient from a patient association to check for understanding and relevance to the Delphi results.

The questionnaire was then submitted to ten patients for linguistic validation and cognitive debriefing. The time-to-complete was noted. The questionnaire was then reviewed by the investigating centers to obtain the final version.

### Translation

The original French questionnaire was translated into English through three independent forward translations (French to English) followed by two independent back translations (English to French), with reconciliation of the translated texts [26].

### Validation

#### Participants

Patients included in the validation study were recruited by 12 secondary or tertiary care rheumatology centers in France, including 2 private practice centers and a patient association (ANDAR, Association Nationale de Défense contre l’Arthrite Rhumatoïde, Paris, France). In addition, six participating centers were asked to test the reproducibility and the other six to test the sensitivity to change by including patients who were scheduled to participate in an educational session, after completing the questionnaire.

The inclusion criteria checked by the rheumatologist or the rheumatology nurse were: patients aged ≥ 18 years, with RA according to ACR/EULAR classification criteria [27], followed up in out-patient or in-patient care, able to complete a questionnaire in French. Exclusion criteria were conditions that could alter the patients’ understanding such as cognitive impairment and psychiatric disorders.

#### Data collection

A variety of data were collected at inclusion: socio-demographics (age, sex, family status, education level, socioprofessional status (SPS) categorized according to French classification of SPS: higher SPS corresponded to craftsperson, merchant and company head, senior managers and intellectual profession and lower SPS were farmer, intermediate professions, employees, and others without professional activities, disease and treatment characteristics (disease duration, current treatment, non-pharmacological treatment), type of follow-up and each patient’s information sources. Several self-administered questionnaires were completed: Rheumatoid Arthritis Impact of Disease (RAID) score [28], Arthritis Helplessness Index (AHI) [29], General Self-Efficacy scale (GSE) [30], and Beliefs about Medication Questionnaire (BMQ) [31]. The rheumatologist or rheumatology nurse reported his or her opinion of the patient’s level of knowledge on the disease and its treatments using a numeric analog scale.

### Statistics

Sample size was determined according to COSMIN guidelines (https://www.cosmin.nl/). Rules-of-thumb for number of subjects needed for internal consistency vary from four to 10 subjects per variable, with a minimum number of 100 subjects to ensure stability of the variance–covariance matrix whereas, at least 50 subjects were necessary for reproducibility to highlight an intra-class correlation coefficient or a Cohen’s kappa agreement at least 0.70.

Statistical analysis was performed using Stata 15 (StataCorp LP, College Station, TX) with two-sided type-I error set at 5%. Continuous data were expressed as mean and standard deviation or as median and [interquartile range] according to statistical distribution (assumption of normality assessed using the Shapiro–Wilk test). Categorical parameters were expressed as number of patients and associated percentages. In addition to these descriptive statistics, we also addressed the following psychometric properties [32, 33]. Acceptability was assessed based on data quality which was considered good if less than 5% of data was missing for each item/question. Internal consistency was determined using Kuder–Richardson’s alpha coefficient calculated from the good/bad responses (i.e., considering the “I don’t know” responses as bad). A commonly accepted rule of thumb for describing internal consistency α is as follows: *α* ≥ 0.9 is excellent, 0.9 > *α* ≥ 0.8 is good, 0.8 > *α* ≥ 0.7 is acceptable, 0.7 > *α* ≥ 0.6 is questionable, 0.6 > *α* ≥ 0.5 is poor, and 0.5 < *α* is unacceptable. The following values were calculated: item difficulty (proportion of patients providing the correct answer for an item; noted as “p”), item variance (noted as “p (1-p)”), and item-test correlations (corrected item-test point-biserial correlation coefficients, also termed “discrimination index”) [34, 35]. Sampling adequacy was also evaluated by Kaiser–Meyer–Olkin and Bartlett’s test. Reproducibility was assessed by calculating the strength of agreement (for each item, the percentage of identical answers at test and retest for the same patient) and the kappa coefficient, when taking into account true/false/I do not know responses, and, subsequently, correct/incorrect responses. The kappa coefficient, weighted using quadratic weights as appropriate, was used for categorical data (items) to determine test–retest reliability for each item. For total scores, Lin’s concordance correlation coefficient was estimated. Agreement values were considered, again as per the usual recommendations, as poor (< 0.2), weak (0.2–0.4), moderate (0.4–0.6), substantial (0.6–0.8), or almost perfect (> 0.8) [36]. Reproducibility was tested at a 2-week interval. The patients were asked not to “check” their responses between the 2 assessments. Sensitivity to change was assessed by testing the total questionnaire score and each domain scores before and after one patient face-to-face or patient-group education sessions delivered as part of routine care in the rheumatology departments. The results were expressed as Hedges’ effect size and 95% confidence intervals. The relationships between patient characteristics and knowledge levels were evaluated by univariate analysis. The following statistical tests were carried out: a Student’s *t*-test or Mann–Whitney test to compare groups, and Spearman or Pearson correlation coefficients to analyze relationships between continuous parameters.

## Results

### Questionnaire content

The RAKE (RA Knowledge questionnairE) was obtained as a short form of 32 items corresponding to knowledge considered essential and a long form of 45 items also including knowledge considered useful. The long form contains 6 knowledge domains: knowledge of disease (10 items), pharmacological treatments (14), non-pharmacological treatments (7), comorbidity (1), self-care for pain and fatigue (5), adaptative skills for coping with psychosocial and professional issues and the health care system (8) (Supplementary material 1 and 2). Compared with prior questionnaires, the RAKE contains fewer questions about causes and symptoms. However, it does include the role of tobacco consumption in RA onset, the pharmacological strategy and bDMARDs and one question on comorbidities. In non-pharmacological treatments, physical activity was added to joint protection and the questionnaire mentions adaptative skills such as patients’ pathway, relation with HPs, shared decision making, the value of patient education and professional issues.

### Validation

#### Population

The validation strategy included 130 patients from September 2016 to September 2018. Descriptive data are reported in Table 1.

**Table 1:** Patient characteristics and health professional’ opinions

Gender, woman, *n* (%)	108 (83)
Age, mean ± SD (years)	56.4 ± 2.1
Family status, living alone (vs. living with family), *n* (%) (/124)	39 (31.5)
Level of education, secondary education, *n* (%) (/123)	60 (48.8)
Socio-professional status (higher SPS vs. lower SPS), *n* (%) (/126)	35 (27.8)
Disease duration, median [IQR] (years) (/127)	9 [4; 23]
Current treatments	
NSAIDs, *n* (%)	33 (25.4)
Analgesics, *n* (%)	49 (37.7)
Glucocorticoids, *n* (%)	34 (26.2)
DMARDs, *n* (%)	106 (81.5)
Methotrexate, *n* (%)	79 (60.8)
bDMARDs, *n* (%)	82 (63.1)
Physiotherapy, *n* (%)	36 (27.7)
How well informed is your patient about his/her disease? (/10), mean ± SD (/109)	6.2 ± 2.2
How well informed is your patient about his/her treatment? (/10), mean ± SD (/109)	6.3 ± 2.2
Where did you find information about your disease or treatment?	
From caregivers, *n* (%)	
General practitioner	53 (40.8)
Rheumatologist in a private practice	44 (33.9)
Rheumatologist in hospital	100 (76.9)
Therapeutic education sessions	33 (25.4)
Nurse	17 (13.1)
Other information sources, *n* (%)	
Internet	61 (46.9)
Patient associations	34 (26.2)
Brochures, booklets	50 (38.5)
RAID^a^ (0–10), mean ± SD	4.2 ± 2.2
AHI^5a^ (5–20), mean ± SD	AHI^5 a^ (5–20), mean ± SD
GSE^6b^ (10–40), mean ± SD	28.6 ± 9.0
BMQ^b^ Necessity (5–25), mean ± SD	20.6 ± 4.7
BMQ^a^ Concerns (5–25), mean ± SD	14.4 ± 5.2

*NSAIDs* non-steroidal anti-inflammatory drugs, *DMARDs* disease-modifying anti-rheumatic drugs, *bDMARDs* biological DMARDs, *RAID* Rheumatoid Arthritis Impact of Disease score, *AHI* Arthritis Helplessness Index, *GSE* General Self-Efficacy scale, *BMQ* Beliefs about Medication Questionnaire

^a^High score means bad score

^b^ High score means good score

#### Acceptability

Number of missing data per item was < 5% and total rate of missing data was 1.2% indicating good acceptability.

#### Total score and scores by domains

The scoring ranged from 0 to 100. Mean total score was 72.8 ± 17.8 on the long-form RAKE and 71.3 ± 17.4 on the short-form RAKE. Table 2 reports the responses domain-by-domain. Scores tended to be higher in adaptive skills and lower in comorbidities and self-care.

**Table 2:** RAKE score by domain and the corresponding questions in the 45-item (long-form) and 32-item (short-form) questionnaires

Long-form score (45 items) (%)	72.8 ± 17.8^a^
Short-form score (32 items) (%)	71.3 ± 17.4
Disease knowledge (Q1–8, Q 33–34) (%)	80 [60; 90]
Pharmacological treatments (Q9–19, Q35–37) (%)	79 [57; 86]
Non pharmacological treatments (Q20–24, Q38–39) (%)	71 [57; 100]
Comorbidity (Q25) (%)	54 (41.5%)
Self-care (Q26–28, Q40–41) (%)	80 [60; 80]
Adaptive skills (Q29–32, Q42–45) (%)	88 [75; 100]

Score 0–100

^a^According to their statistical distribution, results for long-form score and short-form score were expressed using mean ± standard deviation, whereas scores for domains were expressed as median [IQR], except for the comorbidities domain which was expressed using number of patients and percentage as it corresponded to only one question

#### *Scores per questions* (Fig. 1)

**Fig. 1:**
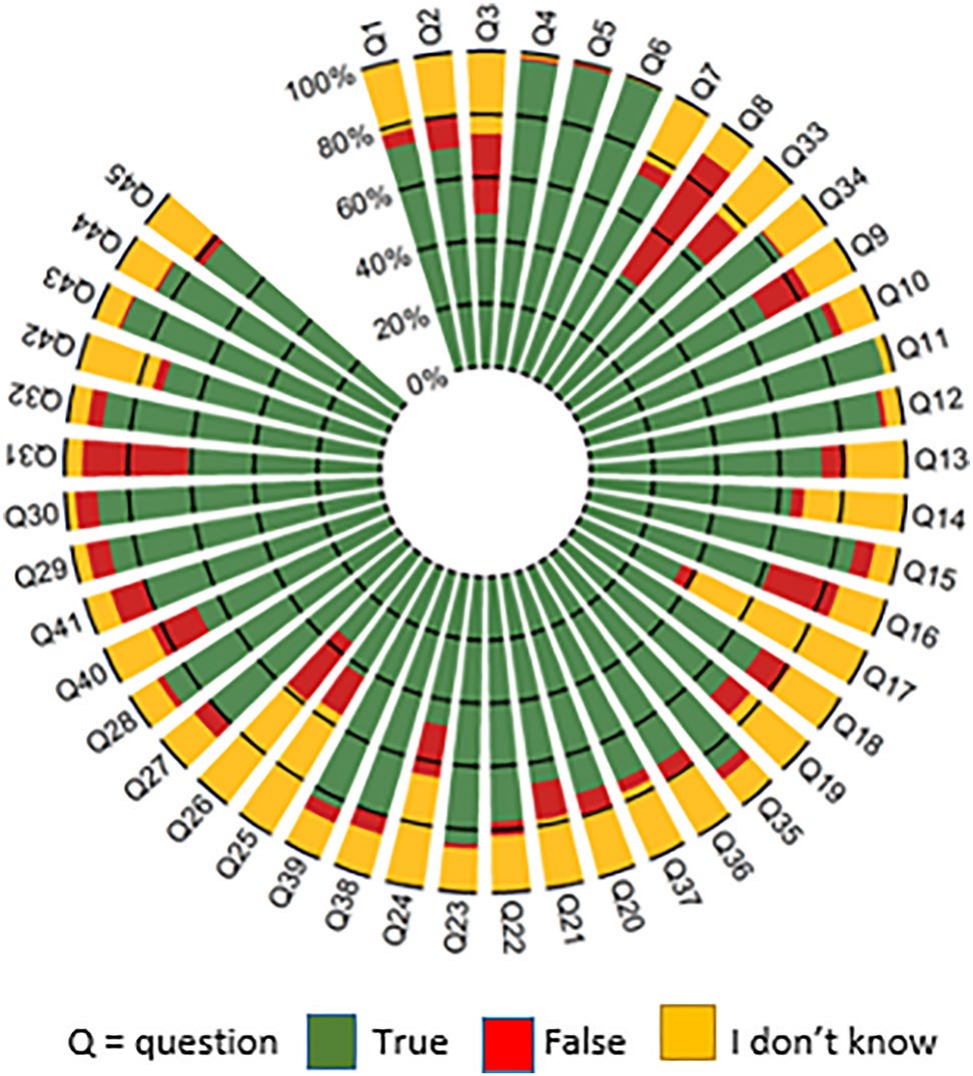
Question-by-question distribution of responses

The rate of “I don’t know” responses ranged from 1% (Q6, RA can cause fatigue) to 59% (Q17, NSAIDs should be stopped if stools turn black). Six questions had a > 50% rate of correct responses, i.e., role of tobacco consumption in RA onset (Q3), diet in RA (Q 24), (NSAIDs (Q17), increased cardiovascular risk in RA (Q25) and preventive use of painkillers before physical activity (Q26). A ≥ 85% correct response rate was found for 13 questions, covering symptoms (3 questions), therapeutic strategies (2), multidisciplinary follow-up (2), professional activity, monitoring of treatment monitoring, stopping cortisone, fatigue, patient involvement and patient associations.

#### Internal validation

The Kuder–Richardson alpha coefficient was 0.90 for the long-form RAKE and 0.85 for the short-form RAKE, indicating excellent internal consistency.

The correlation between items and total long-form RAKE score (“item-retest correlation”) varied between 0.01 and 0.57. The correlations between domains and total long-form the RAKE score are reported in Supplementary material 3. The correlation coefficient between long-form score and short-form RAKE score was excellent at 0.98.

Sampling adequacy was evaluated by Kaiser–Meyer–Olkin (equals 0.7) and Bartlett’s test (*p* < 0.001).

#### Reproducibility

Reproducibility was assessed in 72 subjects. Lin’s concordance correlation coefficient was satisfactory for long-form RAKE score, i.e., 0.86 [95% confidence interval 0.80; 0.92] and excellent for short-form RAKE score, i.e., 0.87 [0.81; 0.92]. The concordances by questions and domains are reported in Supplementary material 4.

#### External validity

External validity was confirmed by a statistically significant correlation with the degree of information the patient had about his or her disease and treatment as gauged by the doctor or nurse (respectively, *r* = 0.55, *r* = 0.58) as well as a significant correlation with the patients’ level of education (*p* < 0.001) (Table 4). RAKE score was weakly correlated with BMQ necessity (*r* = 0.24, *p* = 0.005) (positive correlation), BMQ concerns (r = -0.24, p = 0.006) (negative correlation) and GSE (*r* = 0.27, *p* = 0.003) (positive correlation). RAKE score was not correlated with RAID score (*r* = − 0.16, *p* = 0.08) and AHI (*r* = − 0.18, *p* = 0.04).

The clinically relevant relationships between the domains and the RAID, GSE AHI and BMQ scores (necessity and concerns) were investigated. There was a small but statistically significant correlation (*r* = 0.19, *p* = 0.03) between knowledge on pharmacological treatment and BMQ necessity and an inverse moderate significant correlation between knowledge on pharmacological treatment and BMQ concerns (*r* = − 0.29, *p* = 0.001). GSE score had a small but significant correlation with the domains of disease knowledge, pharmacology treatment, non-pharmacological treatment, and adaptive skills.

#### Sensitivity to change

**Fig. 2:**
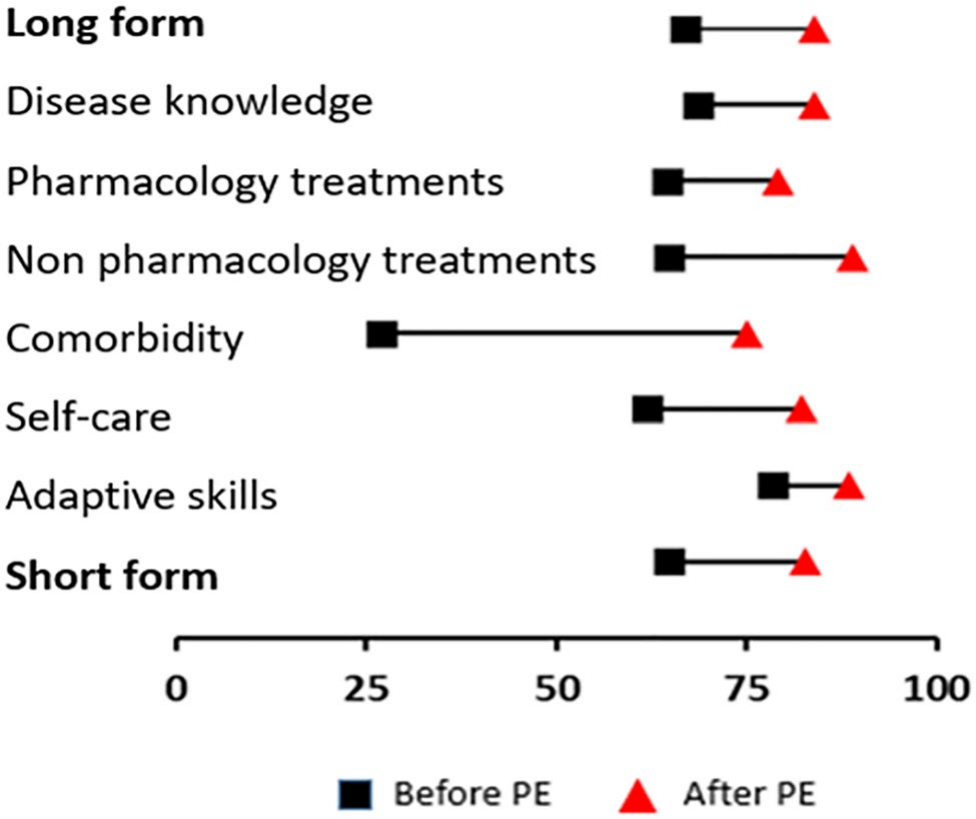
Correct response rate before and after patient education (%) in the 45-item (long-form) questionnaire

Sensitivity to change was measured in 54 patients. There was a statistically significant difference in total score between the two assessment times: 66.8 ± 16.4 vs. 83.8 ± 12.7 (*p* < 0.001), representing an effect size of 1.14 [95% CI: 0.73; 1.55]. Domain-by-domain results are reported in Fig. 2 and Table 3. The domains with higher progression were comorbidity, non-pharmacological treatments, and self-care.

**Table 3:** Correct response rate before and after patient education (%) in the 45-item (long-form) questionnaire

	Before PE	After PE
Long form	66.8	83.8
Disease knowledge	68.5	83.9
Pharmacology treatments	64.5	79.1
Non pharmacology treatments	64.8	89
Comorbidity	27	75
Self-care	61.9	82.3
Adaptive skills	78.4	88.5
Short form	64.8	82.8

*PE* patient education

Table 4 reports the factors associated with knowledge levels in the long-form RAKE. This was a high correlation between better knowledge and higher levels of education, and a low positive correlation between knowledge and female gender and longer disease duration. Older patients had lower knowledge. There was a moderate correlation between the actual knowledge level of patients and the HP’s estimate. Patients with the highest response rate had information sourced from patient associations, brochures and booklets, or education sessions. For these three categories, there was a significant difference between patients with and without access to these sources.

**Table 4:** Knowledge factors in long-form RAKE questionnaire score

Sex		
Female (*n* = 108)	75.7 ± 15.8	0.005
Male (*n* = 22)	60.4 ± 20.7	
Age	*r* = − 0.18	0.04
Family status		
Alone (*n* = 39)	73.0 ± 15.4	0.98
Not alone (*n* = 85)	73.1 ± 18.6	
Grade level		
> High school (*n* = 63)	65.3 ± 18.0	< 0.001
≤ High school (*n* = 60)	81.0 ± 13.8	
Socio-professional status		
Higher socioprofessional status	75.0 [59.4; 84.4]	0.54
Lower socioprofessional status	78.1 [62.5; 87.5]	
Disease duration	*r* = 0.21	0.02
NSAIDs		
No (*n* = 97)	71.2 ± 17.5	0.09
Yes (*n* = 33)	77.5 ± 18.0	
Analgesic pain		
No (*n* = 81)	70.9 ± 17.1	0.12
Yes (*n* = 49)	76.0 ± 18.5	
Cortisone		
No (*n* = 96)	74.4 ± 17.7	0.10
Yes (*n* = 34)	68.4 ± 17.5	
DMARDs, n (%)		
No (*n* = 10)	79.6 ± 15.9	0.27
Yes (*n* = 106)	73.4 ± 17.0	
Methotrexate, n (%)		
No (*n* = 51)	75.3 ± 17.5	0.21
Yes (*n* = 79)	71.2 ± 17.8	
bDMARDs, n (%)		
No (*n* = 32)	73.3 ± 16.2	0.52
Yes (*n* = 82)	75.5 ± 16.9	
Physiotherapy		
No (*n* = 94)	73.3 ± 16.2	0.69
Yes (*n* = 36)	71.7 ± 21.4	
Fitness exercises		
No (*n* = 118)	72.1 ± 17.8	0.15
Yes (*n* = 12)	79.8 ± 16.8	
How well informed is your patient about his/her disease?	*r* = 0.55	< 0.001
How well informed is your patient about his/her treatment?	*r* = 0.58	< 0.001
Where did you find information about your disease or treatment?		
General practitioner, n (%)		
No (*n* = 77)	75.3 ± 18.0	0.05
Yes (*n* = 53)	69.2 ± 16.9	
Rheumatologist in a private practice, *n* (%)		
No (*n* = 86)	73.3 ± 17.1	0.67
Yes (*n* = 44)	71.8 ± 19.2	
Rheumatologist in hospital, *n* (%)		
No (*n* = 30)	68.1 ± 19.6	0.13
Yes (*n* = 100)	74.2 ± 17.0	
Therapeutic education sessions, *n* (%)		
No (*n* = 97)	69.1 ± 17.6	< 0.001
Yes (*n* = 33)	83.6 ± 13.3	
Nurse, n (%)		
No (*n* = 113)	72.5 ± 18.2	0.61
Yes (*n* = 17)	74.6 ± 15.2	
Other information sources: internet, *n* (%)		
No (*n* = 69)	72.7 ± 18.0	0.94
Yes (*n* = 61)	72.9 ± 17.6	
Other information sources: patient associations, *n* (%)		
No (*n* = 96)	66.4 ± 57.8	< 0.001
Yes (*n* = 34)	90.8 ± 8.20	
Other information sources: brochures and booklets, *n* (%)		
No (*n* = 80)	68.1 ± 18.2	< 0.001
Yes (*n* = 50)	80.4 ± 14.2	

## Discussion

This study describes the development and validation of RAKE, a knowledge questionnaire for RA patients. The RAKE showed good acceptability with a low rate of missing responses, good internal and external consistency, adequate test–retest reproducibility, and good sensitivity to change assessed before and following patient education sessions.

The questionnaire was constructed with involvement and input from both healthcare professionals and patients at each stage in the process. A preliminary study had identified knowledge considered essential or useful for the patients, either from the patients’ perspective or in terms of recommendations put into practice by caregivers [13].

The RAKE addresses patients’ needs for knowledge in the era of targeted drugs providing safety messages on pharmacological treatments and particularly bDMARDs [19]. Furthermore, the RAKE has incorporated treatment strategies such as early management, the goal of remission, and shared decision making with the doctors in accordance with international guidelines [1–3].

This study also showed a lack of basic knowledge on widely used medications such as NSAIDs and analgesics, which raises questions over how HPs convey information in practice: despite the information available online [37, 38], patient awareness of side effects remains insufficient [39]. Previous questionnaires were not geared to improving this knowledge as they did not mention cardiovascular side effects or digestive bleeding. The RAKE could, therefore, help to detect gaps in this area, typically to improving monitoring for blood pressure when taking NSAIDs [40].

The study also found that patients are underinformed on the increased risk of cardiovascular disease in RA, despite it being a major comorbidity [41, 42]. In terms of disease knowledge, the RAKE has placed emphasis on practical messages such as the role of tobacco consumption in the onset of RA, which is another factor that many RA patients were unfamiliar with and that has implications for management of the disease [43].

Regarding non-pharmacological treatments, the RAKE has given focus to physical activity rather than just joint protection, which brings it into line with the latest recommendations [44]. The RAKE also contains information and advice on the proper type of exercise and how to manage exercise-related pain and fatigue, and on other self-care issues. These knowledge items scored relatively poorly in this study but were improved following patient education. Many patients did not know that exclusion diet is not recommended in RA, despite it being currently studied [45].

Other domains addressed in the questionnaire include adaptive skills, which had the highest rate of correct responses, notably on patient pathway, multidisciplinary management of RA, and personal or professional matters [46]. This domain is an originality of the RAKE that emerged through participatory input from patients and HPs. The formulation of the questions on these topics proved to be challenge and their statements often seemed banal and their answers intuitive. However, the designers chose to retain these elements, based on the rationale that a knowledge questionnaire is not merely an assessment tool but also an educational tool that facilitates communication between patients and HPs as part of the educational process [4].

Among the factors associated with better knowledge, we identified a younger age and longer disease duration. Recourse to patient associations, brochures and booklets, or therapeutic education sessions was associated with a higher score, as shown in other studies [19]. RAKE score was weakly correlated to beliefs about medication or self-efficacy, which was to be expected as these concepts share complex determinants.

Strengths of this study include the multicentric validation process, notably through recruitment by a patient association and private practice centers, the substantial involvement of patients, the psychometric validation in line with current guidelines, and the simultaneous validation of a short-form RAKE which would be easier to use in current practice. Another strength of this study is that it detects unmet educational needs on important issues such as symptomatic treatments, comorbidities and tobacco consumption. Conversely, the high score on bDMARDs may be due to a recruitment bias by rheumatology departments in the validation stage, where education on safety competencies regarding targeted DMARDs is already part of current practice.

Limitations of this study include a potential cultural bias, since development of the questionnaire resulted from Delphi rounds that were only conducted in France. Moreover, as mentioned above, the extension of the concept of knowledge from cognitive knowledge to a broader range of practical and coping skills [4], although closer to the patients’ perspective, has made it difficult to elaborate discriminative questions. Another limitation is that the RAKE scores were highly correlated with education level, making it less suitable for people with low literacy, which is another limitation. Additional educational strategies for knowledge assessment will be needed for these patients [47]. Finally, one limitation was inherent to the concept of knowledge scale, as management strategies change over time and can make a knowledge questionnaire obsolete within a few years. This is why the RAKE questionnaire should be used as a starting point for patient education and the health professionals are invited, to provide updated information as necessary.

The RAKE questionnaire may be useful in several contexts. It can be valuable for detecting patient needs for education to help manage their disease and their treatment before or during face-to-face or patient-group sessions, and as a way to initiate HP–patient communication. The RAKE may usefully serve to improve the information delivered by HPs and the content covered in education sessions by evaluating the knowledge level of a population of RA patients. It can help to motivate patients to participate in educational programs by helping them understand certain misconceptions or misbeliefs. The RAKE may also help to beneficially assess the efficacy of education interventions in routine practice or in clinical trials.

In conclusion, the RAKE is an updated questionnaire designed to assess patient knowledge in RA. It has good psychometric proprieties and satisfactory reproducibility and sensitivity to change after patient education. Further studies are now needed in other cultural contexts and to explore the factors currently associated with RA knowledge in RA patients.

## Supplementary Information

Below is the link to the electronic supplementary material.

Supplementary file1 (DOCX 27 KB)

Supplementary file2 (DOCX 27 KB)

Supplementary file3 (DOCX 15 KB)

Supplementary file4 (DOCX 40 KB)

Supplementary file5 (DOCX 278 KB)

## Declarations

### Data Sharing

Deidentified participant data will be available upon request by contacting the principal investigator Malory Rodère mrodere@chu-clermontferrand.fr and/or the Data manager Bruno Pereira MD PhD bpereira@chu-clermontferrand.fr. Reuse of data is permitted with a signed data access agreement.
