# Supplementary material for: Development and validation of a self-administered questionnaire measuring essential knowledge in patients with rheumatoid arthritis
Source: Rheumatol Int. 2022 Apr 7;42(10):1785–95. doi: 10.1007/s00296-022-05090-8 (PMC9439984; doi:10.1007/s00296-022-05090-8)
Supplement: Supplementary file 1 — Supplementary file1 (DOCX 27 KB) [file 296_2022_5090_MOESM1_ESM.docx]

**Supplementary material 1: RAKE (english version)**

In this questionnaire, “RA” refers to rheumatoid arthritis.

We will ask you some questions about your rheumatic disease. Please answer the following questions by ticking true or false or I don't know.

| QUESTIONS | True | False | I don’t know |
| --- | --- | --- | --- |
| MAIN list |  |  |  |
| 1. RA is automatically passed on to children. | **□** | **□** | **□** |
| 2. RA is an autoimmune disorder. | □ | □ | □ |
| 3. Smoking increases your chances of developing RA. | □ | □ | □ |
| 4. Joint swelling can be a sign of inflammation in RA. | □ | □ | □ |
| 5.RA pain only appears during daytime. | □ | □ | □ |
| 6. RA can cause fatigue. | □ | □ | □ |
| 7.Joints are more likely to be damaged if they are swollen for a long period of time. | □ | □ | □ |
| 8. A blood test is enough to diagnose RA. | □ | □ | □ |
| 9. The goal of RA management is remission. | □ | □ | □ |
| 10. Steroids/glucocorticoids or non-steroidal anti-inflammatory drugs (NSAIDs) are enough to treat RA. | □ | □ | □ |
| 11. RA disease-modifying drugs must be monitored because of their possible side-effects. | □ | □ | □ |
| 12. RA treatment is the same for all patients. | □ | □ | □ |
| 13. Biologics should be interrupted, if there is infection or fever. | □ | □ | □ |
| 14. Biologics should be interrupted before scheduled surgery. | □ | □ | □ |
| 15. Long term steroids/glucocorticoids can be stopped overnight. | □ | □ | □ |
| 16. Steroids/glucocorticoids are disease-modifying drugs. | □ | □ | □ |
| 17. Non-steroidal anti-inflammatory drugs (NSAIDs) should be stopped if stools are black. | □ | □ | □ |
| 18.The only painkiller (analgesic) authorized for RA is acetaminophen/paracetamol. | □ | □ | □ |
| 19. When taking painkillers (analgesics), you should stop anti-inflammatory drugs. | □ | □ | □ |
| 20. If your joints are stiff in the morning, you should not do exercises by yourself. | □ | □ | □ |
| 21. Physical activity helps reduce fatigue in RA. | □ | □ | □ |
| 22. Splints/braces can be useful during flares. | □ | □ | □ |
| 23. Suitable footwear can limit pain and deformities. | □ | □ | □ |
| 24. A specific diet excluding certain food types is necessary.in RA. | □ | □ | □ |
| 25. There is a higher risk of heart disease (e.g., heart attack) in RA. | □ | □ | □ |
| 26.Painkillers (analgesics) can be taken before physical activity to prevent pain. | □ | □ | □ |
| 27. Fatigue is only due to lifestyle. | □ | □ | □ |
| 28. To manage fatigue, all you have to do is take medication. | □ | □ | □ |
| 29. A person with RA must be followed-up by both the rheumatologist and the general practitioner. | □ | □ | □ |
| 30. RA is too complex to ask your doctor questions about. | □ | □ | □ |
| 31. In general, family and friends understand the pain and fatigue due to RA. | □ | □ | □ |
| 32. People with RA have to stop all professional activity. | □ | □ | □ |
| 33. RA will always worsen with time. | □ | □ | □ |
| 34. RA heals in about ten years. | □ | □ | □ |
| 35. When disease-modifying drugs are started at time of diagnosis, joints are less likely to be damaged. | □ | □ | □ |
| 36. A low-salt and calcium-rich diet can reduce the side-effects of steroids/glucocorticoids. | □ | □ | □ |
| 37. Acetaminophen/paracetamol can be concealed in fever and cold medicine. | □ | □ | □ |
| 38. Regular fitness exercises are beneficial in RA. | □ | □ | □ |
| 39. You must not do sports when you have RA. | □ | □ | □ |
| 40. Once the pain has settled in, painkillers (analgesics) may be less effective. | □ | □ | □ |
| 41. Pain linked to inflammation goes away with rest. | □ | □ | □ |
| 42. Patients education can help cope with the disease. | □ | □ | □ |
| 43. RA management may require other health professionals, in addition to doctors, e.g., physical therapist. | □ | □ | □ |
| 44. Patient associations can be a helpful resource. | □ | □ | □ |
| 45. Recognition of disabled worker status often allows working environment adjustments. | □ | □ | □ |

RAKE Scoring

Each question obtains one point for the RIGHT answer (indicated in the column) and 0 for another answer or a missing answer.

| QUESTIONS | Right answer (worth one point) | Patients score |
| --- | --- | --- |
| **Main list** |  |  |
| 1. RA is automatically passed on to children. | False |  |
| 2. RA is an autoimmune disorder. | True |  |
| 3. Smoking increases your chances of developing RA. | True |  |
| 4. Joint swelling can be a sign of inflammation in RA. | True |  |
| 5.RA pain only appears during daytime. | False |  |
| 6. RA can cause fatigue. | True |  |
| 7.Joints are more likely to be damaged if they are swollen for a long period of time. | True |  |
| 8. A blood test is enough to diagnose RA. | False |  |
| 9. The goal of RA management is remission. | True |  |
| 10. Steroids/glucocorticoids or non-steroidal anti-inflammatory drugs (NSAIDs) are enough to treat RA. | False |  |
| 11. RA disease-modifying drugs must be monitored because of their possible side-effects. | True |  |
| 12. RA treatment is the same for all patients. | False |  |
| 13. Biologics should be interrupted, if there is infection or fever. | True |  |
| 14. Biologics should be interrupted before scheduled surgery. | True |  |
| 15. Long term steroids/glucocorticoids can be stopped overnight. | False |  |
| 16. Steroids/glucocorticoids are disease-modifying drugs. | False |  |
| 17. Non-steroidal anti-inflammatory drugs (NSAIDs) should be stopped if stools are black. | True |  |
| 18.The only painkiller (analgesic) authorized for RA is acetaminophen/paracetamol. | False |  |
| 19. When taking painkillers (analgesics), you should stop anti-inflammatory drugs. | False |  |
| 20. If your joints are stiff in the morning, you should not do exercises by yourself. | False |  |
| 21. Physical activity helps reduce fatigue in RA. | True |  |
| 22. Splints/braces can be useful during flares. | True |  |
| 23. Suitable footwear can limit pain and deformities. | True |  |
| 24. A specific diet excluding certain food types is necessary.in RA. | False |  |
| 25. There is a higher risk of heart disease (e.g., heart attack) in RA. | True |  |
| 26.Painkillers (analgesics) can be taken before physical activity to prevent pain. | True |  |
| 27. Fatigue is only due to lifestyle. | False |  |
| 28. To manage fatigue, all you have to do is take medication. | False |  |
| 29. A person with RA must be followed-up by both the rheumatologist and the general practitioner. | True |  |
| 30. RA is too complex to ask your doctor questions about. | False |  |
| 31. In general, family and friends understand the pain and fatigue due to RA. | False |  |
| 32. People with RA have to stop all professional activity. | False |  |
| **Additional list** |  |  |
| 33. RA will always worsen with time. | False |  |
| 34. RA heals in about ten years. | False |  |
| 35. When disease-modifying drugs are started at time of diagnosis, joints are less likely to be damaged. | True |  |
| 36. A low-salt and calcium-rich diet can reduce the side-effects of steroids/glucocorticoids. | True |  |
| 37. Acetaminophen/paracetamol can be concealed in fever and cold medicine. | True |  |
| 38. Regular fitness exercises are beneficial in RA. | True |  |
| 39. You must not do sports when you have RA. | False |  |
| 40. Once the pain has settled in, painkillers (analgesics) may be less effective. | True |  |
| 41. Pain linked to inflammation goes away with rest. | False |  |
| 42. Patients education can help cope with the disease. | True |  |
| 43. RA management may require other health professionals, in addition to doctors, e.g., physical therapist. | True |  |
| 44. Patient associations can be a helpful resource. | True |  |
| 45. Recognition of disabled worker status often allows working environment adjustments. | True |  |
| Sum of points |  |  |
| Total score/100: (sum x 100) /45 |  |  |
