# Supplementary material for: Development and validation of a self-administered questionnaire measuring essential knowledge in patients with rheumatoid arthritis
Source: Rheumatol Int. 2022 Apr 7;42(10):1785–95. doi: 10.1007/s00296-022-05090-8 (PMC9439984; doi:10.1007/s00296-022-05090-8)
Supplement: Supplementary file 2 — Supplementary file2 (DOCX 27 KB) [file 296_2022_5090_MOESM2_ESM.docx]

**Supplementary material 2 RAKE (French version)**

Dans ce questionnaire le mot « polyarthrite » fait référence à la polyarthrite rhumatoïde

Nous allons vous poser quelques questions sur votre rhumatisme. Veuillez répondre aux questions suivantes par vrai ou faux ou je ne sais pas.

| QUESTIONS | Vrai | Faux | Je ne sais pas |
| --- | --- | --- | --- |
| **LISTE PRINCIPALE** |  |  |  |
| 1. On transmet automatiquement la polyarthrite à ses enfants. | □ | □ | □ |
| 2. La polyarthrite est due à un dérèglement de l’immunité. | □ | □ | □ |
| 3. La polyarthrite est favorisée par le tabac. | □ | □ | □ |
| 4. L’inflammation peut se manifester par un gonflement des articulations. | □ | □ | □ |
| 5. Les douleurs de la polyarthrite apparaissent seulement dans la journée. | □ | □ | □ |
| 6. La polyarthrite peut provoquer de la fatigue. | □ | □ | □ |
| 7. Les articulations ont plus de risques de s’abimer si elles restent gonflées longtemps. | □ | □ | □ |
| 8. La prise de sang suffit pour poser le diagnostic de polyarthrite. | □ | □ | □ |
| 9. L’objectif du traitement dans la polyarthrite est d’obtenir une rémission. | □ | □ | □ |
| 10. La cortisone ou les anti-inflammatoires non stéroïdiens (AINS) sont suffisants pour traiter une polyarthrite. | □ | □ | □ |
| 11. Les traitements de fond de la polyarthrite nécessitent tous une surveillance du fait de leurs effets indésirables possibles. | □ | □ | □ |
| 12. Le traitement de la polyarthrite est le même pour tous les patients. | □ | □ | □ |
| 13. Il faut arrêter la biothérapie en cas d’infection ou de fièvre. | □ | □ | □ |
| 14. Il faut arrêter la biothérapie avant une intervention chirurgicale prévue. | □ | □ | □ |
| 15. La cortisone prise au long cours peut être arrêtée du jour au lendemain. | □ | □ | □ |
| 16. La cortisone est un traitement de fond. | □ | □ | □ |
| 17. Il faut arrêter les anti-inflammatoires non stéroïdiens (AINS) si les selles deviennent noires. | □ | □ | □ |
| 18. Le seul anti-douleur (antalgique), autorisé pour la polyarthrite est le paracétamol. | □ | □ | □ |
| 19. Si on prend des anti douleurs (antalgiques), les anti-inflammatoires doivent être arrêtés. | □ | □ | □ |
| 20. En cas de raideur des articulations le matin, il est contre-indiqué de faire soi-même des exercices. | □ | □ | □ |
| 21. L'activité physique aide à diminuer la fatigue de la polyarthrite. | □ | □ | □ |
| 22. Les orthèses de repos (ou attelles) peuvent être utiles en cas de poussée. | □ | □ | □ |
| 23. Le choix des chaussures peut limiter les douleurs et les déformations des pieds. | □ | □ | □ |
| 24. La polyarthrite nécessite un régime excluant certains aliments | □ | □ | □ |
| 25. Quand on a polyarthrite, on a plus de risques de faire une maladie cardiaque (par exemple un infarctus). | □ | □ | □ |
| 26. Les anti-douleurs (antalgiques) peuvent être pris en prévention, avant les exercices physiques. | □ | □ | □ |
| 27. La fatigue est uniquement due au mode de vie. | □ | □ | □ |
| 28. Pour gérer sa fatigue, il suffit de prendre un médicament. | □ | □ | □ |
| 29. Une personne atteinte de polyarthrite doit être suivie en commun par son rhumatologue et son médecin traitant. | □ | □ | □ |
| 30. La polyarthrite est une maladie trop compliquée pour poser des questions à son médecin. | □ | □ | □ |
| 31. De façon générale, la famille et les proches comprennent bien la douleur et la fatigue de la polyarthrite. | □ | □ | □ |
| 32. Il faut cesser toute activité professionnelle lorsqu’on a une polyarthrite. | □ | □ | □ |
| **LISTE COMPLEMENTAIRE** |  |  |  |
| 33. La polyarthrite s’aggrave toujours et de façon continue. | □ | □ | □ |
| 34. La polyarthrite guérit en une dizaine d’années. | □ | □ | □ |
| 35. Lorsque le traitement de fond est commencé dès le diagnostic, les articulations ont moins de risque de s’abimer. | □ | □ | □ |
| 36. Un régime peu salé et riche en calcium peut diminuer les inconvénients de la cortisone. | □ | □ | □ |
| 37. Le paracétamol peut se cacher dans certains médicaments contre la fièvre ou le rhume. | □ | □ | □ |
| 38. Faire régulièrement des exercices de gymnastique est bénéfique quand on a une polyarthrite. | □ | □ | □ |
| 39. Le sport est contre indiqué quand on a une polyarthrite. | □ | □ | □ |
| 40. Lorsqu’on laisse la douleur s’installer, les anti-douleurs (antalgiques) sont parfois moins efficaces. | □ | □ | □ |
| 41. Une douleur liée à l’inflammation disparait au repos. | □ | □ | □ |
| 42. On peut mieux vivre sa maladie grâce à l’éducation thérapeutique. | □ | □ | □ |
| 43. La prise en charge de la polyarthrite peut nécessiter l’intervention d’autres professionnels de santé, en plus des médecins, par exemple un kinésithérapeute. | □ | □ | □ |
| 44. Il existe des associations de patients pouvant être une ressource et une aide. | □ | □ | □ |
| 45. La reconnaissance de travailleur handicapé permet souvent l’adaptation des conditions de travail. | □ | □ | □ |

RAKE Score

**Chaque question est cotée 1 point si la réponse est juste et 0 si la réponse est fausse, “ je ne sais pas”, réponse manquante ou autre réponse.**

| QUESTIONS | Bonne réponse  (1 point) | Points du patient |
| --- | --- | --- |
| **Liste principale** |  |  |
| 1. On transmet automatiquement la polyarthrite à ses enfants. | Faux |  |
| 2. La polyarthrite est due à un dérèglement de l’immunité. | Vrai |  |
| 3. La polyarthrite est favorisée par le tabac. | Vrai |  |
| 4. L’inflammation peut se manifester par un gonflement des articulations. | Vrai |  |
| 5. Les douleurs de la polyarthrite apparaissent seulement dans la journée. | Faux |  |
| 6. La polyarthrite peut provoquer de la fatigue. | Vrai |  |
| 7. Les articulations ont plus de risques de s’abimer si elles restent gonflées longtemps. | Vrai |  |
| 8. La prise de sang suffit pour poser le diagnostic de polyarthrite. | Faux |  |
| 9. L’objectif du traitement dans la polyarthrite est d’obtenir une rémission. | Vrai |  |
| 10. La cortisone ou les anti-inflammatoires non stéroïdiens (AINS) sont suffisants pour traiter une polyarthrite. | Faux |  |
| 11. Les traitements de fond de la polyarthrite nécessitent tous une surveillance du fait de leurs effets indésirables possibles. | Vrai |  |
| 12. Le traitement de la polyarthrite est le même pour tous les patients. | Faux |  |
| 13. Il faut arrêter la biothérapie en cas d’infection ou de fièvre. | Vrai |  |
| 14. Il faut arrêter la biothérapie avant une intervention chirurgicale prévue. | Vrai |  |
| 15. La cortisone prise au long cours peut être arrêtée du jour au lendemain. | Faux |  |
| 16. La cortisone est un traitement de fond. | Faux |  |
| 17. Il faut arrêter les anti-inflammatoires non stéroïdiens (AINS) si les selles deviennent noires. | Vrai |  |
| 18. Le seul anti-douleur (antalgique), autorisé pour la polyarthrite est le paracétamol. | Faux |  |
| 19. Si on prend des anti douleurs (antalgiques), les anti-inflammatoires doivent être arrêtés. | Faux |  |
| 20. En cas de raideur des articulations le matin, il est contre-indiqué de faire soi-même des exercices. | Faux |  |
| 21. L'activité physique aide à diminuer la fatigue de la polyarthrite. | Vrai |  |
| 22. Les orthèses de repos (ou attelles) peuvent être utiles en cas de poussée. | Vrai |  |
| 23. Le choix des chaussures peut limiter les douleurs et les déformations des pieds. | Vrai |  |
| 24. La polyarthrite nécessite un régime excluant certains aliments. | Faux |  |
| 25. Quand on a polyarthrite, on a plus de risques de faire une maladie cardiaque (par exemple un infarctus). | Vrai |  |
| 26. Les anti-douleurs (antalgiques) peuvent être pris en prévention, avant les exercices physiques. | Vrai |  |
| 27. La fatigue est uniquement due au mode de vie. | Faux |  |
| 28. Pour gérer sa fatigue, il suffit de prendre un médicament. | Faux |  |
| 29. Une personne atteinte de polyarthrite doit être suivie en commun par son rhumatologue et son médecin traitant. | Vrai |  |
| 30. La polyarthrite est une maladie trop compliquée pour poser des questions à son médecin. | Faux |  |
| 31. De façon générale, la famille et les proches comprennent bien la douleur et la fatigue de la polyarthrite. | Faux |  |
| 32. Il faut cesser toute activité professionnelle lorsqu’on a une polyarthrite. | Faux |  |
| **Liste complémentaire** | Faux |  |
| 33. La polyarthrite s’aggrave toujours et de façon continue. | Faux |  |
| 34. La polyarthrite guérit en une dizaine d’années. | Faux |  |
| 35. Lorsque le traitement de fond est commencé dès le diagnostic, les articulations ont moins de risque de s’abimer. | Vrai |  |
| 36. Un régime peu salé et riche en calcium peut diminuer les inconvénients de la cortisone. | Vrai |  |
| 37. Le paracétamol peut se cacher dans certains médicaments contre la fièvre ou le rhume. | Vrai |  |
| 38. Faire régulièrement des exercices de gymnastique est bénéfique quand on a une polyarthrite. | Vrai |  |
| 39. Le sport est contre indiqué quand on a une polyarthrite. | Faux |  |
| 40. Lorsqu’on laisse la douleur s’installer, les anti-douleurs (antalgiques) sont parfois moins efficaces. | Vrai t |  |
| 41. Une douleur liée à l’inflammation disparait au repos. | Faux |  |
| 42. On peut mieux vivre sa maladie grâce à l’éducation thérapeutique. | Vrai |  |
| 43. La prise en charge de la polyarthrite peut nécessiter l’intervention d’autres professionnels de santé, en plus des médecins, par exemple un kinésithérapeute. | Vrai |  |
| 44. Il existe des associations de patients pouvant être une ressource et une aide. | Vrai |  |
| 45. La reconnaissance de travailleur handicapé permet souvent l’adaptation des conditions de travail. | Vrai |  |
| Somme des points |  | ………… |
| Score total /100 : (somme x 100)/45 |  | …………. |
