# Supplementary material for: Development and validation of a self-administered questionnaire measuring essential knowledge in patients with rheumatoid arthritis
Source: Rheumatol Int. 2022 Apr 7;42(10):1785–95. doi: 10.1007/s00296-022-05090-8 (PMC9439984; doi:10.1007/s00296-022-05090-8)
Supplement: Supplementary file 3 — Supplementary file3 (DOCX 15 KB) [file 296_2022_5090_MOESM3_ESM.docx]

**Supplementary material 3.** The correlations between domains and total long-form the RAKE score.

|  | **Long-form** | *Disease knowledge* | *Pharmacology treatments* | *Non pharmacology treatments* | *Comorbidity* | *Self-care* | *Adaptive skills* | **Short-form** |
| --- | --- | --- | --- | --- | --- | --- | --- | --- |
| **Long-form** | 1 |  |  |  |  |  |  |  |
| *Disease knowledge* | 0.828 | 1 |  |  |  |  |  |  |
| *Pharmacology treatments* | 0.867 | 0.643 | 1 |  |  |  |  |  |
| *Non pharmacology treatments* | 0.815 | 0.565 | 0.634 | 1 |  |  |  |  |
| *Comorbidity* | 0.514 | 0.376 | 0.413 | 0.401 | 1 |  |  |  |
| *Self-care* | 0.710 | 0.473 | 0.533 | 0.549 | 0.390 | 1 |  |  |
| *Adaptive skills* | 0.727 | 0.545 | 0.510 | 0.547 | 0.273 | 0.459 | 1 |  |
| **Short-form** | 0.982 | 0.810 | 0.894 | 0.801 | 0.515 | 0.676 | 0.657 | 1 |

*Correlations ranged from 0.51 to 0.86, with highest correlations (> 0.80) for domains of disease knowledge, pharmacological treatments and non-pharmacological treatments. The domains of comorbidity, self-care and adaptive skills had a lower correlation with the total long-form the RAKE score (between 0.51 and 0.72).*
