# Supplementary material for: Development and validation of a self-administered questionnaire measuring essential knowledge in patients with rheumatoid arthritis
Source: Rheumatol Int. 2022 Apr 7;42(10):1785–95. doi: 10.1007/s00296-022-05090-8 (PMC9439984; doi:10.1007/s00296-022-05090-8)

**Supplementary material 4.**

Figure a: Lin’s concordance coefficient by domains and for long-form and short-form scores.


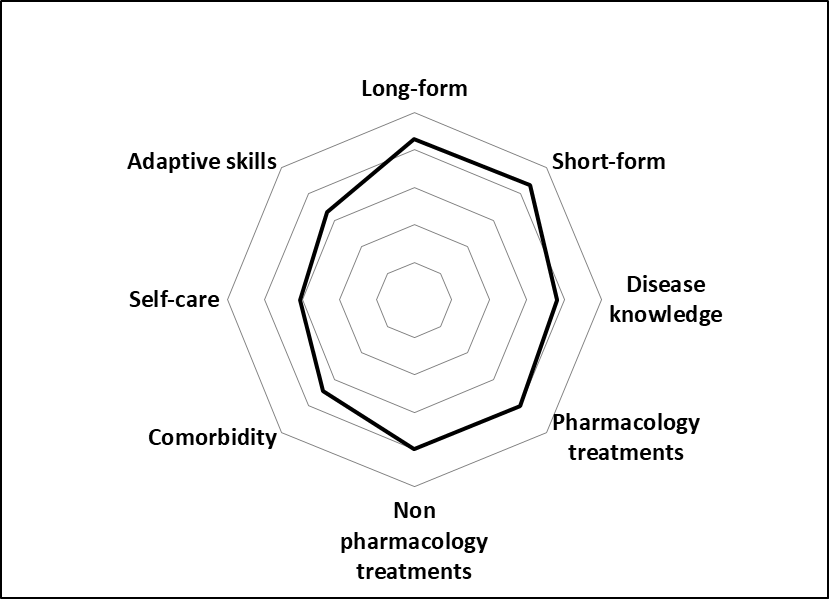

Supplement: Supplementary file 4 — Supplementary file4 (DOCX 40 KB) [file 296_2022_5090_MOESM4_ESM.docx]
