# Supplementary material for: Development and validation of a self-administered questionnaire measuring essential knowledge in patients with rheumatoid arthritis
Source: Rheumatol Int. 2022 Apr 7;42(10):1785–95. doi: 10.1007/s00296-022-05090-8 (PMC9439984; doi:10.1007/s00296-022-05090-8)
Supplement: Supplementary file 5 — Supplementary file5 (DOCX 278 KB) [file 296_2022_5090_MOESM5_ESM.docx]

Figure b: Kappa concordance coefficient by questions


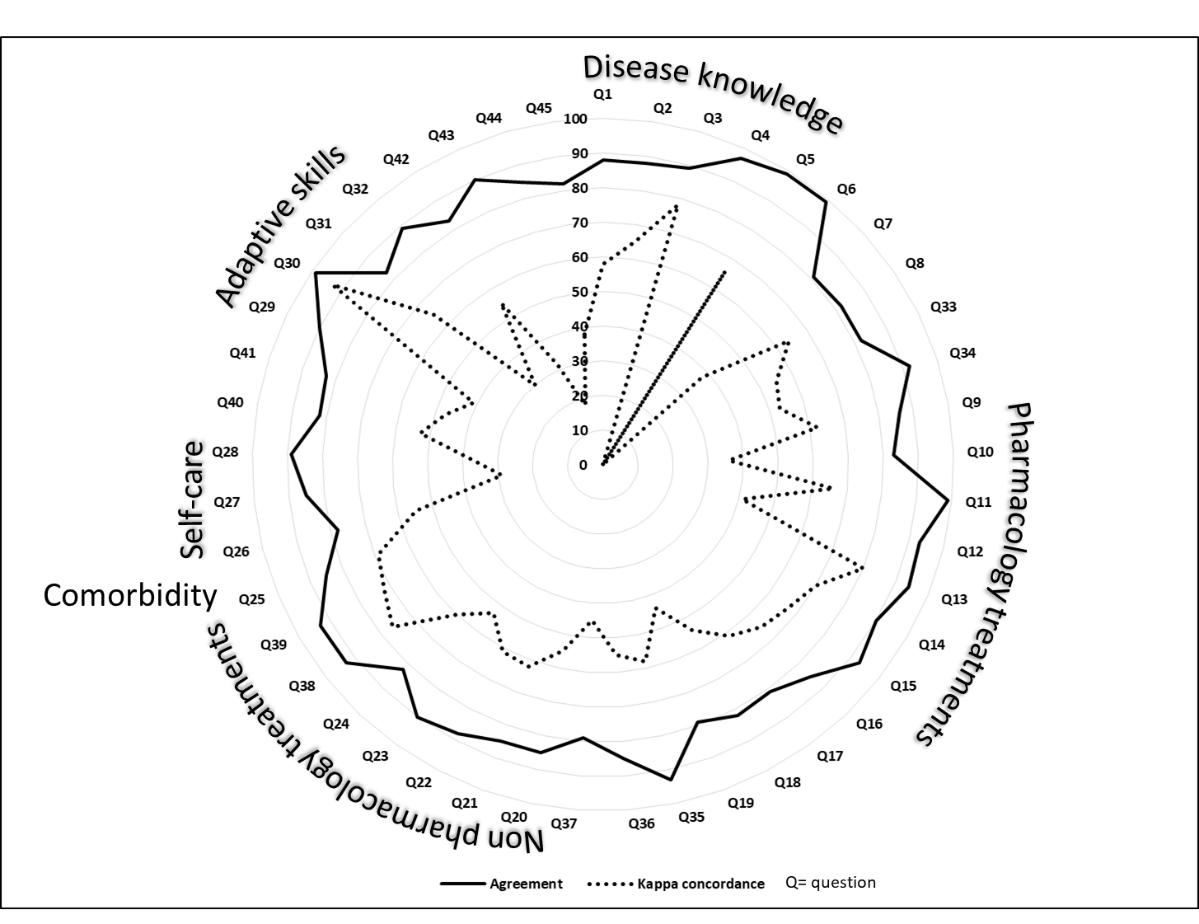


*Concerning the domains, concordance coefficient varied between 0.61 [0.47; 0.75] for self-care and 0.66 [0.54; 0.78] for adaptative skills, and 0.80 [0.72; 0.88] for both dimensions associated with treatments.*

*Of the 45 questions, 16 had a concordance rate of more than 90% and 26 had a value between 80% and 90%. For only 3 questions (Q19, Q26, Q37, all concerning analgesics), the concordance rate was less than 80% (79%, 78% and 79% respectively).*
